# MicroRNA-137 Inhibits Cancer Progression by Targeting Del-1 in Triple-Negative Breast Cancer Cells

**DOI:** 10.3390/ijms20246162

**Published:** 2019-12-06

**Authors:** Soo Jung Lee, Jae-Hwan Jeong, Seung Hee Kang, Jieun Kang, Eun Ae Kim, Jeeyeon Lee, Jin Hyang Jung, Ho Yong Park, Yee Soo Chae

**Affiliations:** 1 Department of Oncology/Hematology, School of Medicine, Kyungpook National University, Kyungpook National University Chilgok Hospital, Daegu 41404, Korea; majestio@hanmail.net; 2 Cell and Matrix Research Institute, School of Medicine, Kyungpook National University, Daegu 41944, Korea; jamesjh@hanmail.net (J.-H.J.); majestio03@gmail.com (S.H.K.); hermione-j@hanmail.net (J.K.); llmllmllmllx@naver.com (E.A.K.); 3 Department of Breast & Thyroid Surgery, School of Medicine, Kyungpook National University, Kyungpook National University Chilgok Hospital, Daegu 41404, Korea; drjeeyeon@naver.com (J.L.); jjh01@knu.ac.kr (J.H.J.); 4 Joint Institute for Regenerative Medicine, Kyungpook National University, Daegu 41944, Korea

**Keywords:** biomarker, *Del-1*, developmental endothelial locus-1, miR-137, triple-negative breast cancer

## Abstract

MicroRNAs (miRNAs) can be used to target a variety of human malignancy by targeting their oncogenes or tumor suppressor genes. The developmental endothelial locus-1 (Del-1) might be under miRNA regulation. This study investigated microRNA-137 (miR-137) function and Del-1 expression in triple-negative breast cancer (TNBC) cells and tissues. *Del-1* mRNA and miRNA-137 levels were determined via qRT-PCR in breast cancer cells (MDA-MB-231, MCF7, SK-BR3, and T-47D) and tissues from 30 patients with TNBC. The effects of miR-137 on cell proliferation, migration, and invasion were determined using MTT assays, wound healing, and Matrigel transwell assays. The luciferase reporter assay revealed direct binding of miR-137 to the 3′-UTR of *Del-1*. miR-137 inhibited cell proliferation, migration, and invasion of MDA-MB-231 cells. Among the 30 TNBC specimens, miR-137 was downregulated and Del-1 level in plasma was significantly elevated relative to normal controls. It is concluded that miR-137 regulates Del-1 expression in TNBC by directly binding to the *Del-1* gene and cancer progression. The results implicate miR-137 as a new therapeutic biomarker for patients with TNBC.

## 1. Introduction

Many recent advances in breast cancer diagnosis and treatment have relied on the development of new imaging modalities and the discovery of novel small-molecule chemotherapies. However, most of these therapies are limited to hormone-responsive or human epidermal growth factor receptor 2 (HER2)-overexpressing breast cancer subtypes. New molecular targets or biomarkers are needed for the triple-negative subtype, especially given its unfavorable outcomes.

Developmental endothelial locus-1 (Del-1), also referred to as epidermal growth factor-like repeats, and discoidin domains-3 (Edil-3), is widely expressed in breast cancer. Moreover, Del-1 on circulating extracellular vesicles was remarkably elevated in the plasma of two separate cohorts of breast cancer patients, and levels returned to normal after curative surgery, which suggested that Del-1 could be a novel diagnostic marker for early breast cancer [1]. Among several mechanisms proposed for the effects of Del-1 on cancer development and progression, perhaps the most supported is the idea that Del-1 alters the p53 pathway and thereby promotes tumor growth and inhibits apoptosis [2,3].

While direct alterations of target genes involved in the apoptosis or proliferation of breast cancer cells can occur and accumulate in target cells via a complex and multi-step process, indirect control mechanisms include altered target gene expression. MicroRNAs (miRNAs) are 19–25 nucleotide long, single-stranded, non-coding RNA molecules that regulate target gene expression by translational repression or degradation of mRNAs. Although the specific targets and biological functions of miRNAs are not completely known, miRNAs are crucial in regulating (as either tumor suppressors or oncogenes) the expression of genes controlling diverse cellular and metabolic pathways in cancer cells; some miRNAs have been associated with specific cancers [4,5,6,7].

We hypothesized that specific miRNAs may play a role in Del-1 expression in tumor tissues or plasma and could, thus, be novel therapeutic targets together with the *Del-1* gene or protein itself. Therefore, we aimed to investigate the function of a candidate miRNA, miR-137, in Del-1 expression in TNBC cells and tissues. 

## 2. Results

### 2.1. Negative Correlation between miR-137 and Del-1 Expression in TNBC Cell Lines

**Figure 1:**
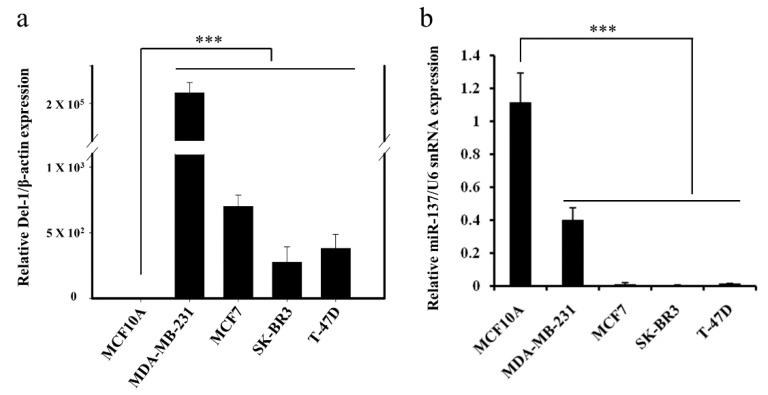
Expression of *Del-1* mRNA and miR-137 in breast epithelial and cancer cell lines. (**a**) Comparison of the *Del-1* mRNA level between breast epithelial and breast cancer cell lines. Compared to MCF10A breast epithelial cells, *Del-1* mRNA was highly expressed in all breast cancer cell lines. Expression was particularly high in MDA-MB-231 triple-negative breast cancer cells. (**b**) The expression of miR-137 was downregulated in all breast cancer cells. *** *p* < 0.001.

Recently, we reported the detection of significant amounts of exosomal Del-1 in plasma from patients with breast cancer and the abundant expression of Del-1 protein in various breast cancer cell lines [8]. In the present study, we searched for miRNAs targeting *Del-1* and clarified their inter-relationship in breast cancer. Based on a bioinformatics search using three well-known prediction algorithm programs, miR-137 was predicted and then selected as a potential miRNA targeting *Del-1* (Table 1). Since in silico analyses remain speculative, both direct interaction between miRNA and target mRNA and their precise binding sites within *Del-1* were further determined in vitro. Real-time quantitative reverse transcriptase- polymerase chain reaction (qRT-PCR) was conducted to confirm the expression of *Del-1* mRNA and miR-137 in the breast cancer cell lines. Consistent with previously reported western blot results [8], *Del-1* mRNA was remarkably overexpressed in MDA-MB-231 TNBC cells when compared to MCF7, SK-BR3, and T-47D breast cancer cells and MCF10A normal breast epithelial cells (Figure 1a). The miR-137 levels were significantly lower in various breast cancer cell lines compared to MCF10A (Figure 1b), indicating a possible association between miR-137 and Del-1 expression in TNBC. Thus, further analyses were performed using MDA-MB-231 cells to investigate the function of miR-137.

### 2.2. MiR-137 Directly Targets the 3′-UTR of Del-1 to Inhibit Del-1 Expression

**Figure 2:**
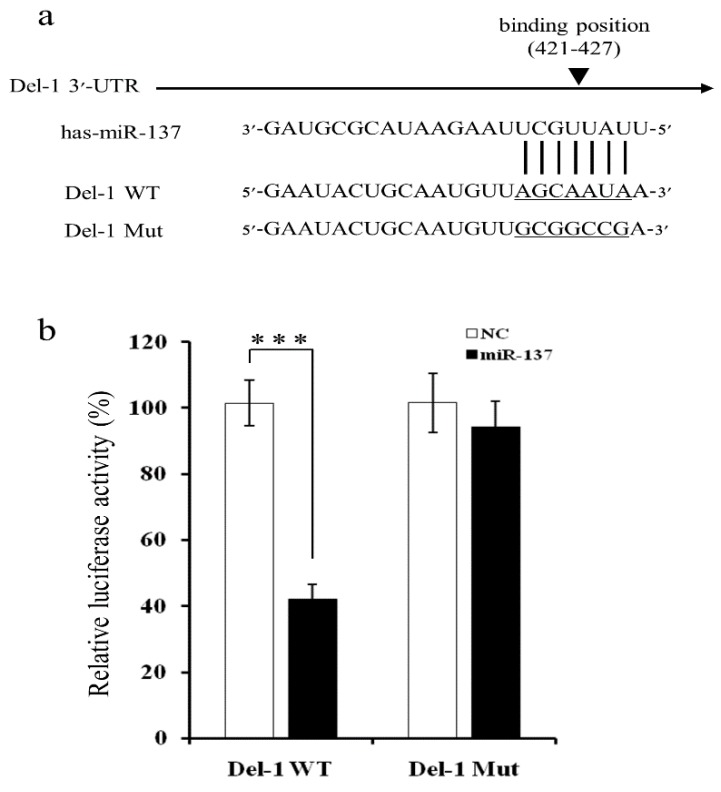
Identification of target sites for miR-137. (**a**) The putative target site for miR-137 was predicted to be located within the 3′-untranslated region (UTR) of *Del-1* mRNA at nucleotides 421-427. (**b**) Luciferase reporter assay evaluation of the interaction between miR-137 and the 3′-UTR of *Del-1* mRNA. MDA-MB-231 cells were transfected with luciferase constructs containing the wild-type (Del-1 WT) or a mutated (Del-1 Mut) 3′-UTR of *Del-1* mRNA and miRNA mimic or negative control. Luciferase activity was determined 24 h after transfection. Data represent the mean ± SD of three independent experiments. *** *p* < 0.001.

Given that bioinformatic analyses predicted that putative target sites for miR-137 were located within the 3′-untranslated region (UTR) of *Del-1* mRNA (Figure 2a), mutants at the predicted binding sites were constructed to further investigate the interaction with miR-137. The plasmids were transfected with luciferase vectors containing either wild-type (WT) or a mutant-type (Mut) *Del-1* 3′-UTR, and either a synthetic miR-137 mimic or a negative control. When MDA-MB-231 cells were co-transfected with WT *Del-1* 3′-UTR and a miR-137 mimic, the miR-137 mimic significantly reduced luciferase activity by approximately 60%, when compared to co-transfection of WT *Del-1* 3′-UTR and a negative control (Figure 2b). Luciferase activity of the Mut *Del-1* 3′-UTR vector did not change following co-transfection with the miR-137 mimic, suggesting that *Del-1* was indeed the target of miR-137.

### 2.3. miR-137 Regulation of Endogenous Del-1 Expression

**Figure 3:**
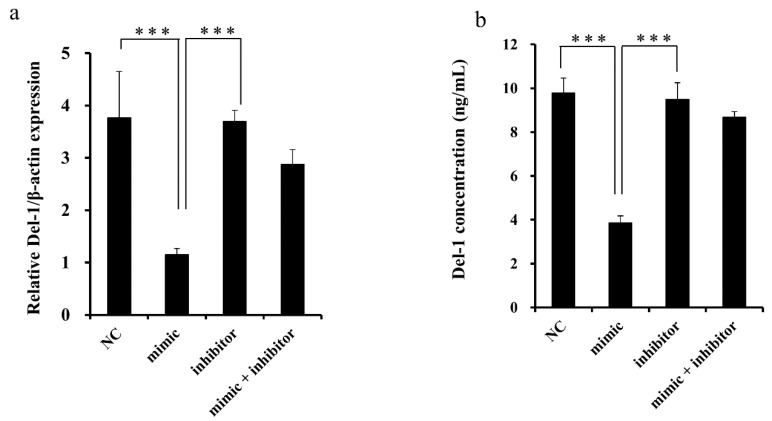
Del-1 expression following miR-137 overexpression and knockdown. (**a**) Relative *Del-1* mRNA expression was evaluated by qRT-PCR 48 h after transfection with miR-137 mimic, inhibitor, or mimic plus inhibitor. Overexpression of miR-137 in MDA-MB-231 cells by transfection of miR-137 mimic resulted in a significant reduction in *Del-1* mRNA transcription, which was rescued by transfection of miR-137 inhibitor or mimic plus inhibitor. (**b**) Concentration of Del-1 protein was measured in the culture medium by ELISA 48 h after transfection with miR-137 mimic, inhibitor, or mimic plus inhibitor in MDA-MB-231 cells. A significant decrease in Del-1 protein expression was observed in the culture medium following transfection with the miR-137 mimic as compared with the control group (NC). This effect was abrogated when cells were transfected with the miR-137 inhibitor or mimic plus inhibitor. The bar graph depicts the mean ± SD. *** *p* < 0.001.

To confirm the functional impact of miR-137 on Del-1 expression, *Del-1* mRNA and protein levels were determined in MDA-MB-231 breast cancer cells using qRT-PCR and enzyme-linked immunosorbent assay (ELISA) after transient transfection with the mimic or inhibitor of miR-137. Overexpression of miR-137 by transfection of the miR-137 mimic resulted in a significant reduction of *Del-1* mRNA, which was rescued by transfection with the miR-137 inhibitor (Figure 3). These results suggested that miR-137 suppresses the translational activity of the *Del-1* gene by targeting the binding site in the 3′-UTR of *Del-1* mRNA, thereby affecting Del-1 secretion from the breast cancer cells.

### 2.4. miR-137 Inhibits MDA-MB-231 Cell Proliferation, Migration, and Invasion

**Figure 4:**
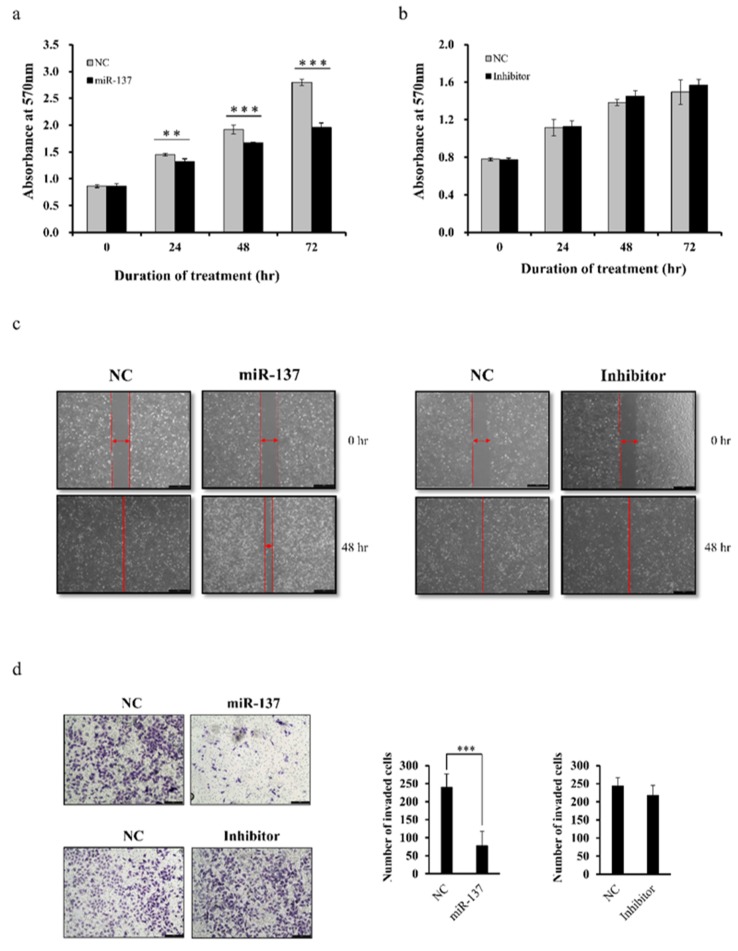
Proliferation, migration, and invasion assays following the overexpression and knockdown of miR-137. (**a**,**b**) In the MTT assay, the proliferation of MDA-MB-231 cells was impaired following the transfection of miR-137 mimic and was completely rescued by the miR-137 inhibitor. (**c**) Wound scratch assay showing that cell migration was suppressed when MDA-MB-231 breast cancer cells were transfected with the miR-137 mimic as compared to the scramble mimic. This effect was reversed by the miR-137 inhibitor. Scale bar = 200 µm. (**d**) Matrigel transwell cell invasion assay showed that ectopic overexpression of miR-137 significantly suppressed cell invasion, while the miR-137 inhibitor did not change the invasion ability of the MDA-MB-231 cells. Scale bar = 200 µm. ** *p* < 0.01, *** *p* < 0.001.

Given that *Del-1* is the target of miR-137 and promotes the progression of MDA-MB-231 breast cancer cells, assays were performed to investigate the proliferation, migration, and invasion of MDA-MB-231 transfected with either mimics or inhibitors of miR-137 to confirm whether miR-137 reverses the oncogenic effect of Del-1 in TNBC. The 3-(4,5-dimethylthiazol-2-yl)-2,5-diphenyltetrazolium bromide (MTT) assay revealed that inhibition of Del-1 expression by transfection of MDA-MB-231 cells with the miR-137 mimic decreased cell proliferation (Figure 4a), whereas the inhibitors had no effect on cell proliferation relative to negative controls (Figure 4b). Migration and invasion of MDA-MB-231 cells were significantly inhibited in a scratch wound-healing assay (Figure 4c) and Matrigel transwell assay (Figure 4d), respectively, by ectopic overexpression of miR-137. However, inhibition was completely rescued by the miR-137 inhibitor. In addition, we evaluated the functional effects of miR-137 in another TNBC cell line, Hs578T, to exclude cell-line-dependent effects. Down-regulation of Del-1 inhibited the proliferation, migration, and invasion of Hs578t cells, which showed consistent results with MDA-MB-231 cells (Supplementary Figure S1).Thus, collective data suggested that miR-137 (via Del-1 regulation) plays a tumor-suppressive role during TNBC progression.

### 2.5. Del-1 Expression Regulates Proliferation, Migration, and Invasion of MDA-MB-231 Cells

**Figure 5:**
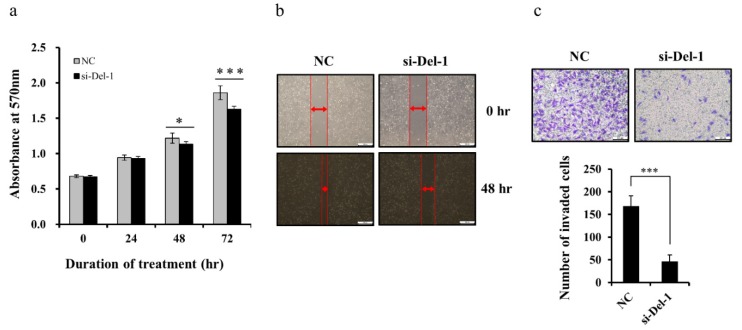
Knockdown of Del-1 suppresses the proliferation, migration, and invasiveness of MDA-MB-231 cells. (**a**) Cell proliferation assay following transfection with si-Del-1 or negative control (NC) siRNA. (**b**) Wound-healing assay results. MDA-MB-231 cells were scratched with a pipette tip and transfected with si-Del-1 or NC, and cultured for 48 h. Scale bar = 200 µm. (**c**) Cell invasion assay of MDA-MB-231 cells after transfection with si-Del-1 or NC siRNA was performed using the transwell assay with Matrigel-coated membranes. The invasiveness of MDA-MB-231 cells was measured after 48 h. Numbers of invasive cells per field were significantly reduced for MDA-MB-231 cells transfected with si-Del-1 versus NC. Data presented as mean ± SD. Scale bar = 200 µm. * *p* < 0.05, *** *p* < 0.001.

**Figure 6:**
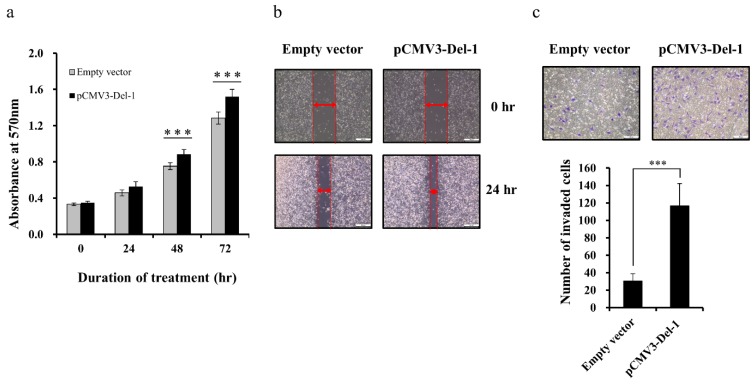
Forced expression of Del-1 accelerates proliferation, migration, and invasiveness of MDA-MB-231 cells. (**a**) Cell proliferation assay following transfection with Del-1 expression vector. (**b**) Wound-healing assay results. MDA-MB-231 cells were scratched with a pipette tip and transfected with Del-1 expression vectors, and then cultured for 24 h. Scale bar = 200 µm. (**c**) Cell invasion assay of MDA-MB-231 cells after transfection with the Del-1 expression vector was performed using the Matrigel transwell assay. The invasiveness of MDA-MB-231 cells was measured after 24 h. Numbers of invasive cells per field increased significantly following transfection with the Del-1 expression vector versus cells transfected with empty vectors. Data are presented as mean ± SD. Scale bar = 200 µm. *** *p* < 0.001.

We previously reported that exosomal Del-1 can promote the invasion of breast cancer cells [8]. To better define the functional role of Del-1 in the tumor progression of TNBC cells, we treated MDA-MB-231 cells with small-interfering RNA (siRNA), which leads to knockdown of Del-1 expression. The efficiency of interference was determined by qRT-PCR and ELISA. Del-1-specific siRNA successfully downregulated *Del-1* mRNA and protein expression in MDA-MB-231 cells (Supplementary Figure S3a,b). The effect of Del-1 downregulation on the proliferation of MDA-MB-231 cells was determined by MTT assay. The proliferation of MDA-MB-231 cells was significantly inhibited by siRNA-mediated downregulation of Del-1 (Figure 5a). In the wound-healing assay, decreased Del-1 expression inhibited MDA-MB-231 cell migration (Figure 5b). Given that invasiveness plays an important role in tumor progression, we conducted transwell assays with Matrigel-coated membranes to evaluate the effects of Del-1 downregulation on the invasiveness of MDA-MB-231 cells. Compared with that of negative controls, invasiveness was significantly reduced following the knockdown of Del-1.(Figure 5c). Moreover, in a transient transfection assay, forced expression of Del-1 increased the proliferation, migration, and invasion of MDA-MB-231 cells (Figure 6; Supplementary Figure S2c,d). In addition, we found consistent results in another TNBC cell line, Hs578T (Supplementary Figure S3). These results indicated that Del-1 may play an important role in the proliferation, migration, and invasion of MDA-MB-231 cells. 

### 2.6. Expression of miR-137 and Del-1 in TNBC Patients

**Figure 7:**
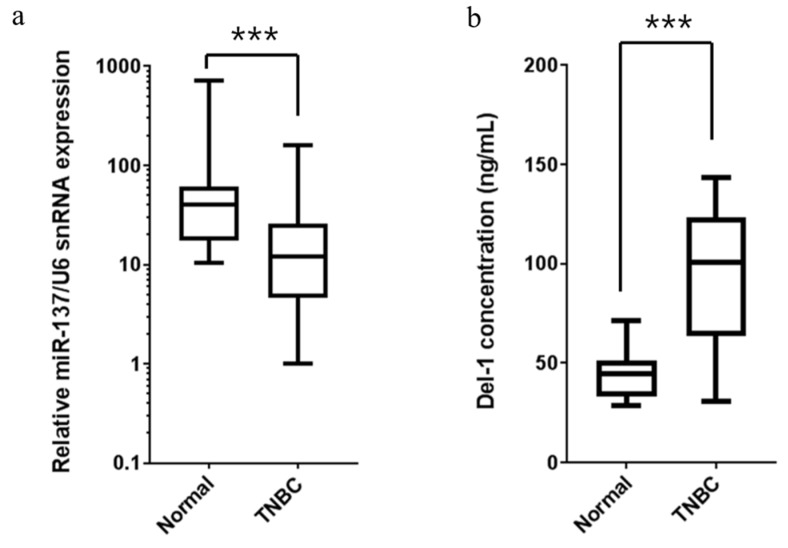
Expressions of miR-137 and Del-1 in patients with triple-negative breast cancer (TNBC). (**a**) Breast tumor tissues from 30 patients with TNBC showed downregulated miR-137 expression, irrespective of patients’ clinical and pathologic characteristics (*n* = 30 for each group). (**b**) Plasma Del-1 levels in TNBC patients detected using ELISA. Plasma Del-1 levels increased in TNBC patients versus normal controls (NC) (*n* = 30 for each group). *** *p* < 0.001.

**Figure 8:**
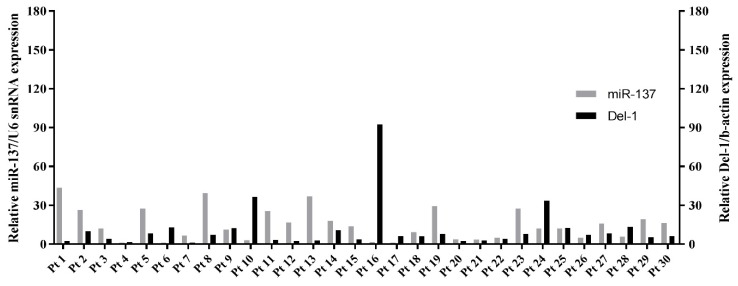
Expressions of miR-137 and Del-1 in patients with triple-negative breast cancer (TNBC). Breast tumor tissues from 30 patients with TNBC showed a trend toward negative correlation between levels of miR-137 and *Del-1* mRNA, irrespective of patients’ clinical and pathologic characteristics (*n* = 30).

To confirm the association between miR-137 and Del-1 expression in TNBC, we measured miR-137 levels in tumor tissues and Del-1 levels in the plasma of 30 consecutive TNBC patients (Figure 7). The miR-137 was significantly downregulated in TNBC tissues as compared to normal controls, irrespective of clinical and pathologic characteristics, including age, tumor size, nodal status, Ki-67 expression, lymph vascular invasion, histologic grade, and germline *BRCA* mutation status (Figure 7a). As expected, plasma Del-1 level was significantly higher in TNBC patients than in normal controls (*p* < 0.0001) (Figure 7b). To confirm our results, we measured miR-137 and Del-1 levels in the human clinical samples, finding a trend toward negative correlation between miR-137 and *Del-1* mRNA levels (Figure 8)

## 3. Discussion

This study found that miR-137 was significantly downregulated in TNBC tissues, plasma, and TNBC cell lines. In addition, *Del-1* was a direct target of miR-137 in TNBC cells; miR-137 directly bound to the 3′-UTR of *Del-1* mRNA. Further, the overexpression of miR-137 inhibited TNBC cell proliferation, invasion, and migration by decreasing Del-1 expression; these effects were rescued by inhibitors of miR-137. Thus, when combined with recent evidence of Del-1 as a diagnostic and potential prognostic marker of breast cancer [8], our results identify novel mRNA/miRNA interactions that contribute to TNBC via a Del-1 mechanism and that represent potential targets for the development of new therapeutic strategies for TNBC.

Del-1 (or Edil-3) was initially identified as an extracellular matrix protein with three N-terminal epidermal growth factor-like domains and discoidin 1-like, or factor V, C domains: C1 and C2. More recently, the involvement of Del-1 in multiple cancers, including lung, hepatocellular, and bladder cancers, has been identified. This suggests a possible relationship between Del-1 and cancer development and progression [3,9,10]. Del-1 may directly affect cancer progression via the p53-related apoptosis pathway [2,3] and via transforming growth factor-beta/extracellular signal-regulated kinase-related epithelial–mesenchymal transition [10,11,12]. Del-1 may also act indirectly by inducing angiogenesis and immune tolerance against tumor cells [13,14,15,16].

Despite the evidence of Del-1-related tumor progression, the regulation of Del-1 expression in cancer cells remains essentially unclear. While the direct alteration of target genes can occur in cells and play a role in apoptosis or proliferation via a complex and multi-step process, indirect control mechanisms, such as altered target gene expression, are also possible. For example, as miRNAs act as post-transcriptional regulators of target gene expression, functioning as either tumor suppressors or oncogenes, specific miRNAs may play a role in Del-1 gene expression and differentiated expression in tumor tissues or plasma. This makes specific miRNAs possible novel therapeutic targets instead of the Del-1 gene or protein. Therefore, using web-based algorithms, we selected miR-137 as a potential regulator of Del-1 expression. We then confirmed that miR-137 was downregulated in breast cancer cell lines and that miR-137 suppressed TNBC progression (by modulating Del-1 expression) by directly targeting the 3′-UTR of *Del-1* mRNA. Consequently, miR-137 may be a new affediagnostic and therapeutic biomarker for TNBC.

The current study provides evidence that miR-137 plays a role in tumor suppression by modulating Del-1 expression in TNBC. Several other studies have reported direct or indirect involvement of miR-137 in suppressing tumor proliferation and progression. Various target genes of miR-137 have been suggested, including *CtBP1* (carboxyl-terminal binding protein 1), *YB-1* (Y-box binding protein-1), *ERRα* (estrogen-related receptor α), and *DCLK1* (doublecortin-like kinase 1). These target genes have been identified in various cancers, including gliomas, melanomas, and colorectal, oral, and ovarian cancers [17,18,19,20,21,22]. However, regardless of the target gene or type of malignancy, miR-137 was consistently downregulated in all cancers in which it was measured. This suggests that complex mechanisms are likely involved in the inhibition of tumor proliferation and progression by miRNAs.

Despite our findings, the correlation of miR-137 with breast cancer has not been fully elucidated in a large cohort or in prospective studies. Moreover, the underlying mechanisms remain unclear. Breast cancer cell lines proved useful in laboratory and preclinical investigations and accurately predicted outcomes for endocrine or anti-HER2 therapeutics in clinical trials, particularly for hormone-responsive or HER2-overexpressed cancer subtypes, respectively. However, TNBC is heterogeneous in its pathologic and molecular features, with several subtypes identified based on the gene expression pattern. Therefore, the limitations of using cell lines for investigating TNBC must be recognized. For example, the MDA-MB-231 cell line used in the current study is the most commonly studied cell line for TNBC, but it was established in the 1970s (before the routine evaluation of hormone receptors and HER2). Given that the role of specific miRNAs is ultimately exclusive to different types of cells or tissues, further studies with different types of TNBC cell line are warranted to validate the exact role of miRNAs in breast cancer. Additionally, several studies indicate miR-137 associated with cell proliferation and invasion asides from controlling Del-1, due to its multiple targets. To delineate whether these effects are either by Del-1 or miR-137, knockout of putative miRNA site from Del-1 is needed [23,24,25].

In summary, our data indicate that miR-137 is involved in Del-1 regulation via binding to *Del-1* mRNA and affects cancer progression by altering Del-1 expression. These findings suggest that miR-137 has a tumor-suppressive role by targeting Del-1 in TNBC. Our results also provide new insights into the potential mechanisms of Del-1-related oncogenesis and reveal the potential of miR-137 as a new regulator and future therapeutic target in TNBC.

## 4. Materials and Methods

### 4.1. Candidate miRNA Selection

The miRNA candidates possibly affecting Del-1 expression were selected from a list created by a bioinformatics search of three web-based algorithms: miRanda (http://www.microrna.org/microrna/home.do), TargetScan (http://www.targetscan.org/vert_71/), and miRDB (http://mirdb.org/miRDB/).

### 4.2. Cell Lines and Cell Culture

The MCF10A human breast epithelial cell line and five breast cancer cell lines (MDA-MB-231, Hs578T, MCF7, SK-BR3, and T-47D) were purchased from the American Type Culture Collection (Manassas, VA, USA). MCF10A cells were maintained in Dulbecco’s Modified Eagle’s medium (DMEM)/F-12 in a 1:1 ratio (Lonza, Walkersville, MD, USA) supplemented with 10% fetal bovine serum (FBS; Gibco, Grand Island, NY, USA), 10 ng/mL epidermal growth factor, 0.5 μg/mL hydrocortisone, 100 ng/mL cholera toxin, and 10 μg/mL insulin. MDA-MB-231, Hs578T, MCF7, and SK-BR3 cells were maintained in DMEM (Gibco, Waltham, MA, USA) supplemented with 10% FBS. T-47D cells were cultured in RPMI (Gibco, Waltham, MA, USA) supplemented with 10% FBS. Hs578T cells were maintained in DMEM (Gibco) supplemented with 10% FBS and 10 μg/mL insulin.

### 4.3. RNA Extraction and Quantitative RT-PCR

Total RNA from the cell lines, including all miRNAs, was isolated from cultured cells using RNAiso Plus (TaKaRa, Shiga, Japan) according to the manufacturer’s instructions. The SuperScript III First-Strand Synthesis System for RT-PCR (Invitrogen, Carlsbad, CA, USA) and TaqMan MicroRNA Reverse Transcription Kit (Applied Biosystems, Foster City, CA, USA) were used for reverse transcription of mRNA and miRNA, respectively. Real-time PCR for relative quantification of *Del-1* expression used the Power SYBR Green PCR Master Mix (Applied Biosystems). The abundance of each miRNA was measured using the TaqMan Universal Master Mix II (Applied Biosystems). The relative amount of each miRNA was normalized according to the amount of U6 small nuclear RNA, while all mRNA data were normalized according to the amount of *β-actin* mRNA. The relative expression levels were calculated using the 2^-ΔΔCt^ method. Each sample was analyzed in triplicate. The specific TaqMan probe sets for miR-137 and U6 were purchased from Applied Biosystems. The primers used for real-time PCR were: *Del-1* (5′-TCGAAGACATTGCACTTTGC-3′ and 5′-ACCCAGAGGCTCAGAACAAC-3′), and *β-actin* (5′-TTGCCGACAGGATGCAGAA-3′ and 5′-GCCGATCCACACGGAGTACT-3′).

### 4.4. miRNA Transfection

The miR-137 mimic and negative control (mimic, inhibitor) were purchased from Sigma-Aldrich Co., LLC (St. Louis, MO, USA). The miR-137 inhibitor was purchased from Ambion (Foster City, CA, USA). Cells were inoculated in a 6-well plate (2 × 10^5^ cells/well) for the wound-healing assay or in a 96-well plate (5 × 10^3^ cells/well) for the MTT cell proliferation assay. After overnight culture, cells were transfected using Lipofectamine RNAiMAX Transfection Reagent (Invitrogen) according to the manufacturer’s instructions.

### 4.5. Plasmid Vector Construction for Luciferase Reporter Assays and Mutagenesis

To construct reporter plasmids containing WT *Del-1* 3′-UTR, a 533-base pair fragment of the *Del-1* 3′-UTR was amplified by PCR using complementary DNA from MDA-MB-231 cells as a template, and then cloned into a pTOP Blunt V2 vector (Enzynomics Co. Ltd., Daejeon, Republic of Korea). The *Del-1* 3′-UTR product was cloned at the 3′-position of the luciferase gene of a pmirGLO Dual-Luciferase miRNA Target Expression Vector (Promega, Madison, WI, USA). To construct plasmids with a Mut *Del-1* 3′-UTR, putative miR-137 target bases within the 3′-UTR of *Del-1* were mutated using a Muta-Direct™ Site-Directed Mutagenesis Kit (iNtRON Biotechnology, Gyeonggi-do, Republic of Korea). All products were confirmed by sequencing.

### 4.6. Dual-Luciferase Reporter Assay

MDA-MB-231 cells were added to wells of 24-well plates (4 × 10^4^ cells/well) with growth medium. After 24h, the cells were co-transfected with 100 ng of the pmirGLO Dual-Luciferase expression construct containing 3′-UTR of *Del-1*, 10 pmol of negative control, or a mimic using the Lipofectamine 3000 Transfection Kit (Invitrogen) according to the manufacturer’s protocol. At 48hafter transfection, cell lysates were collected, and luciferase activities were measured using a Dual-Luciferase Reporter Assay System (Promega, Madison, WI, USA) and normalized to Renilla luciferase activity. All experiments were performed in triplicate.

### 4.7. ELISA

Protein levels of Del-1 in the culture medium of MDA-MB-231 cells and in plasma from patients with TNBC were evaluated by ELISA. Del-1 levels in the culture medium were measured manually using the Human EDIL3 DuoSet ELISA Kit (R&D Systems, Inc., Minneapolis, MN, USA) and an ELISA Start Kit (KOMA Biotech, Inc. Seoul, Republic of Korea) according to manufacturers’ instructions. Six independent experiments were performed.

### 4.8. MTT Assay for Cell Proliferation

Cell proliferation was estimated using an MTT assay (Sigma-Aldrich). Briefly, cells were inoculated at 5 × 10^3^ cells/well in a 96-well plate (200 μL per well). After transfection with 1 pmol of miRNA mimic, inhibitor for each miRNA, or negative control, cells were incubated for 1, 2, or 3 days without medium change. After each time point, 50 μL of MTT (2 mg/mL) was added to the wells, and cells were cultured at 37 °C for a further 4 h. After removal of the medium, 150 μL of dimethyl sulfoxide was added and the solution was mixed for 10 min to dissolve formazan crystals. The optical density was determined at 570 nm. All experiments were conducted in triplicate.

### 4.9. Wound Scratch Assay for Cell Migration

To examine the effects of miR-137 on TNBC cell lines such as MDA-MB-231 and Hs578T cell migration, cells were seeded in 6-well culture plates. After overnight culture, a line was scratched into the cell monolayer using a sterile pipette tip and the medium was changed. Subsequently, the cells were transfected with 25 pmol of negative control, miR-137 mimic, or miR-137 inhibitor and further incubated for 48 h. Three independent experiments were performed.

### 4.10. Transwell Assay for Cell Invasion

For the invasion assay, transwell chambers with 8 μm pores were coated with Matrigel (Corning Inc., Tewksbury, MA, USA) and incubated at 37 °C for 2 h to allow gel solidification. After transfection for 24 h with 25 pmol of negative control, miR-137 mimic, or inhibitor of miR-137, cells were re-suspended in DMEM containing 1% FBS, and 1 × 10^4^ cells were plated in the upper chamber. The lower chamber contained the complete medium supplemented with 10% FBS. After incubation for 48 h, cells on the internal surface of the bottom chamber were collected using a cotton swab, fixed with 2% paraformaldehyde, stained with 0.5% crystal violet, and rinsed with phosphate-buffered saline. Finally, four random views were chosen for each culture well under a light microscope (200×) and the number of invading cells in each view was counted.

### 4.11. Expression of miR-137 and Del-1 in Tissues

To analyze the expression of miRNAs and *Del-1* mRNA in TNBC tissue, tumor samples were acquired from 30 patients with TNBC (stage I–IIIA). Total RNA from the breast tissues was extracted using an RNeasy Lipid Tissue Mini Kit (Qiagen, Hilden, Germany) according to the manufacturer’s instructions. The miRNA expression was measured and analyzed considering clinical and pathologic characteristics, such as age, tumor size, lymph node involvement, histologic grade, lymphovascular invasion, and *BRCA* mutation status. For all experiments with patient samples, patient consent was obtained and relevant ethical guidelines were followed.

### 4.12. Knockdown and Overexpression of Del-1 in MDA-MB-231 Cells

The siRNAs specific to Del-1 and negative control, and that were not homologous to any part of the human genome, were purchased from Santa Cruz Biotechnology, Inc. (Dallas, TX, USA). Cells were inoculated in a 6-well plate (2 × 10^5^ cells/well) or a 96-well plate (5 × 10^3^ cells/well). After culture overnight, cells were transfected using Lipofectamine RNAiMAX reagent (Invitrogen) and 1 pmol siRNA in the 96-well plate or 25 pmol siRNA in the 6-well plate.

For overexpression of Del-1, the full-length *Del-1* gene was cloned into the pCMV3 mammalian expression vector to generate the pCMV3-Del-1 construct, and the empty pCMV3 construct was used as a control. MDA-MB-231 cells in 6-well plates and 96-well plates were plated at a densities of 2 × 10^5^ and 5 × 10^3^ cells/well, respectively, for transfection experiments. Cells were transfected with pCMV3 or pCMV3-Del-1 plasmids (2 μg for the 6-well plates, 100 ng for the 96-well plates) using Lipofectamine 3000 (Invitrogen) for 4 h and were grown for predetermined intervals and assayed as necessary. All transfections were performed in triplicate.

### 4.13. Statistical Analyses

All quantitative experiments were performed at least in triplicate. Data were expressed as mean ± standard deviation (SD) and were analyzed using Student’s *t*-test and Mann-Whitney U test with GraphPad Prism version 7 (GraphPad Software, San Diego, CA, USA). A *p*-value ≤ 0.05 was considered statistically significant.

### 4.14. Ethical Considerations

Our prospective research and sample collection were approved by the institutional review board (IRB) of Kyungpook National University Hospital (#2013-09-009-001). Patient consent forms were approved by the IRB of Kyungpook National University Chilgok Hospital. At the time of recruitment, patients received an information leaflet and consent form regarding storage and collection of biological materials, including blood and tissue samples, and future use of the samples for research purposes.

## Figures and Tables

**Table 1:** Target miRNAs selected by using * three web-based algorithms.

No.	miRNA Name	Predicted Database
1	miR-137	Miranda, TargetScan, miRDB
2	miR-496	Miranda, TargetScan, miRDB
3	miR-361-5P	TargetScan, miRDB
4	miR-425	TargetScan, miRDB
5	miR-9500	TargetScan, miRDB
6	miR-2113	TargetScan, miRDB
7	miR-3685	TargetScan, miRDB
8	miR-3163	TargetScan, miRDB
9	miR-4672	TargetScan, miRDB
10	miR-3662	TargetScan, miRDB

* miRanda (http://www.microrna.org/microrna/home.do), TargetScan (http://www.targetscan.org/vert_71/), and miRDB (http://mirdb.org/miRDB/).

## Supplementary Materials

Supplementary materials can be found at https://www.mdpi.com/1422-0067/20/24/6162/s1.

## Abbreviations

HER2	human epidermal growth factor receptor 2
Del-1	developmental endothelial locus-1
Edil-3	epidermal growth factor-like repeats and discoidin domains-3
WT	wild type
Mut	mutant type
ELISA	enzyme-linked immunosorbent assay
NC	control group
siRNA	small-interfering RNA
TNBC	triple negative breast cancer
SD	standard deviation
