# Supplementary material for: MicroRNA-137 Inhibits Cancer Progression by Targeting Del-1 in Triple-Negative Breast Cancer Cells
Source: Int J Mol Sci. 2019 Dec 6;20(24):6162. doi: 10.3390/ijms20246162 (PMC6941134; doi:10.3390/ijms20246162)
Supplement: Supplementary file 1 [file ijms-20-06162-s001.zip › Supplementary figure and legends (1-3).docx]

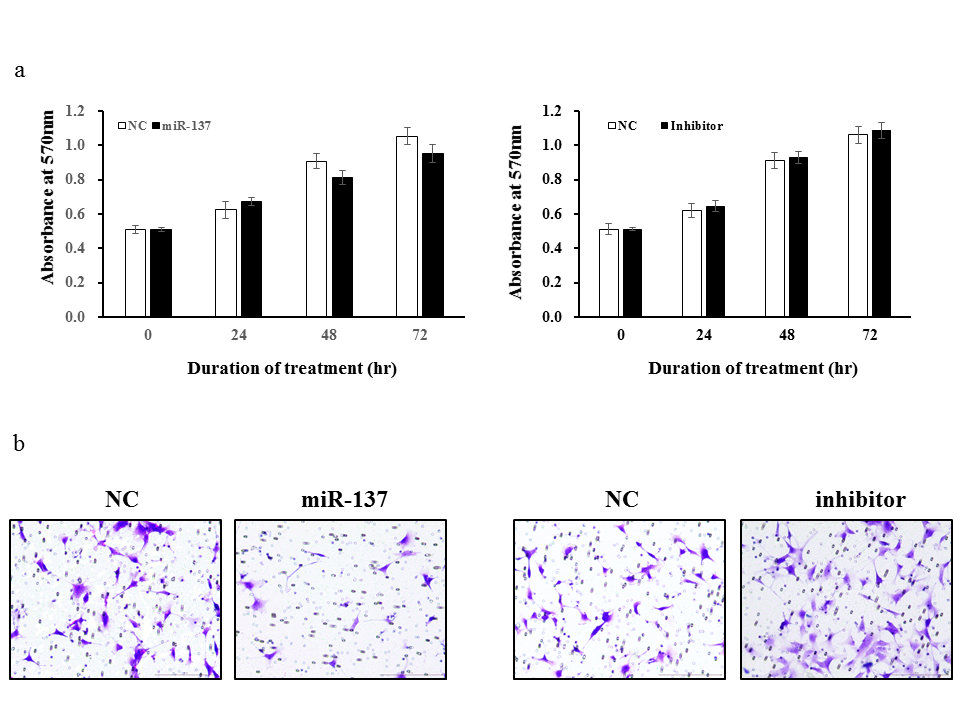
Supplementary figure 1. miR-137 inhibits cell proliferation and invasion of Hs578T cells.

(a) In the MTT assay, the proliferation of Hs578T cells was impaired following the transfection of miR-137 mimic and was completely rescued by the miR-137 inhibitor. (b) Matrigel transwell cell invasion assay showed that ectopic overexpression of miR-137 significantly suppressed cell invasion, while the miR-137 inhibitor did not change the invasion ability of the Hs578T cells.


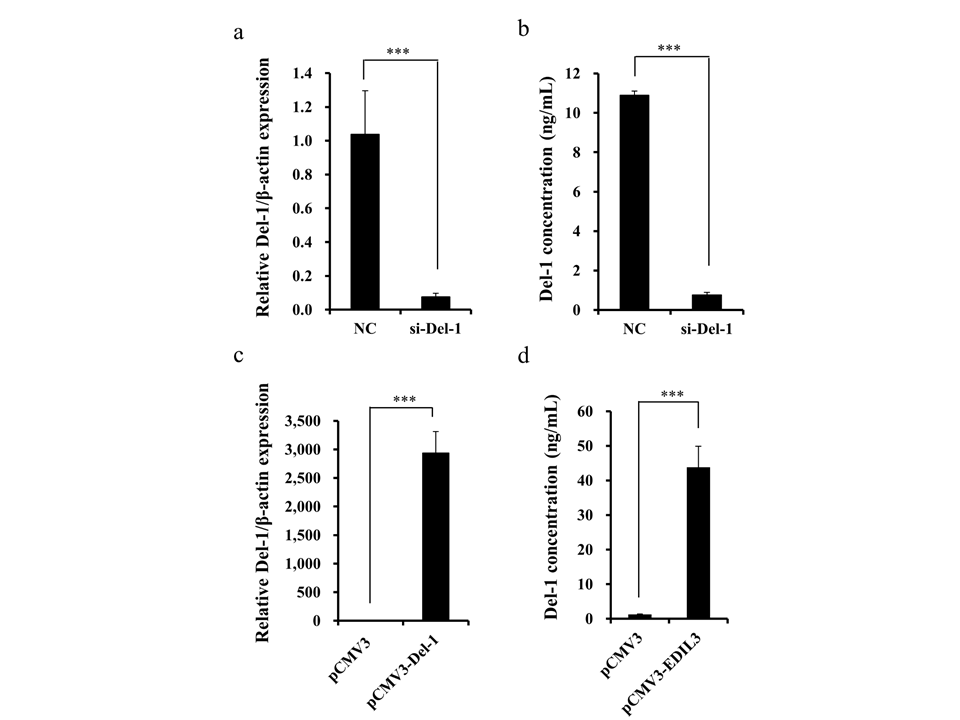


Supplementary figure 2. Downregulation of Del-1 in MDA-MB-231 cells by specific Del-1 siRNA. Knockdown of Del-1 mRNA and protein in the culture medium of MDA-MB-231 cells following si-Del-1 transfection was measured by real-time RT-PCR (a) and ELISA (b) at 48 h after transfection. Upregulation of Del-1 in MDA-MB-231 cells by pCMV3-Del-1 vector. Overexpression of Del-1 mRNA and protein in the culture medium of MDA-MB-231 cells following Del-1 plasmid transformation was measured by real-time RT-PCR (c) and ELISA (d). Data presented as mean ± SD. *** p<0.001.


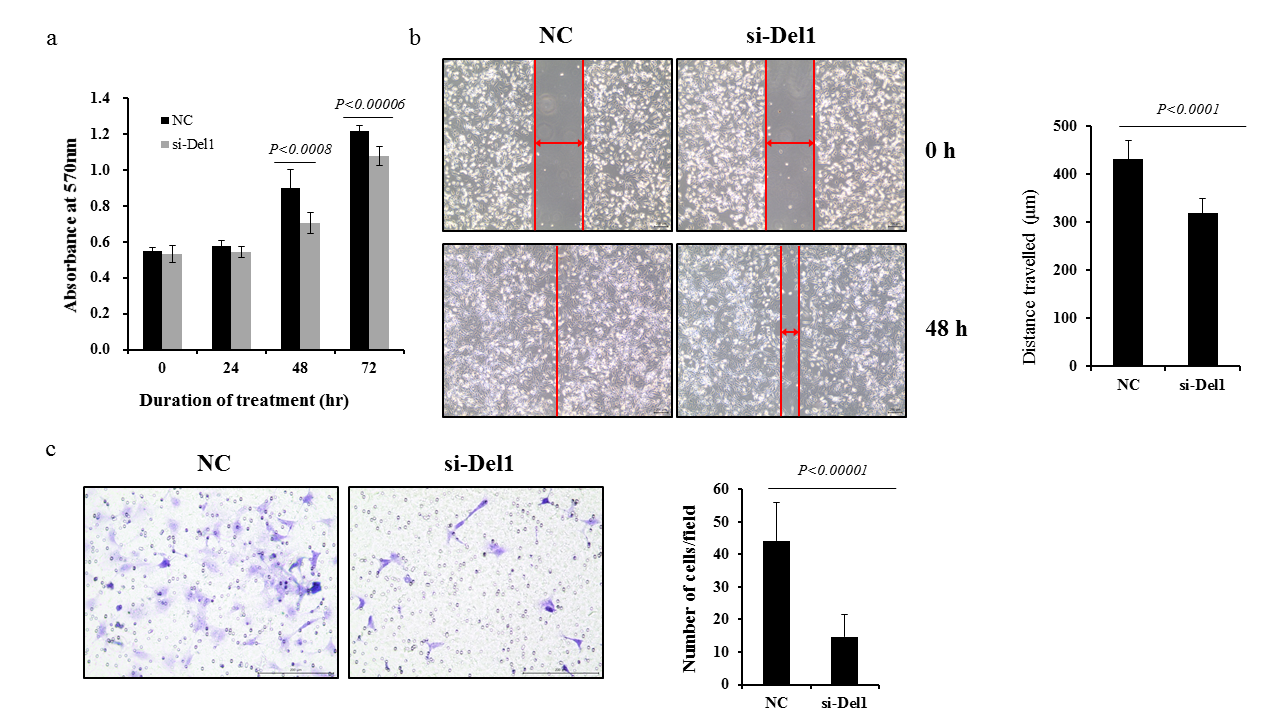


Supplementary figure 3. Knockdown of Del-1 suppresses the proliferation, migration, and invasiveness of Hs578T cells.

(a) This cell proliferation assay was performed following transfection with si-Del-1 or negative control (NC) scrambled siRNA. (b) In this wound-healing assay, Hs578T cells were scratched with a pipette tip and transfected with si-Del-1 or NC siRNA, and then cultured for 48 h. (c) The cell invasion assay with Hs578T cells after transfection with si-Del-1 or NC siRNA was performed using Transwell assays with Matrigel-coated membranes. The invasiveness of Hs578T cells was measured after 48 h; the number of invasive cells per field was significantly reduced in Hs578T cells transfected with si-Del-1 as compared with the NC siRNA group.
