# Supplementary figures and images for: MicroRNA-137 Inhibits Cancer Progression by Targeting Del-1 in Triple-Negative Breast Cancer Cells
Source: Int J Mol Sci. 2019 Dec 6;20(24):6162. doi: 10.3390/ijms20246162 (PMC6941134; doi:10.3390/ijms20246162)

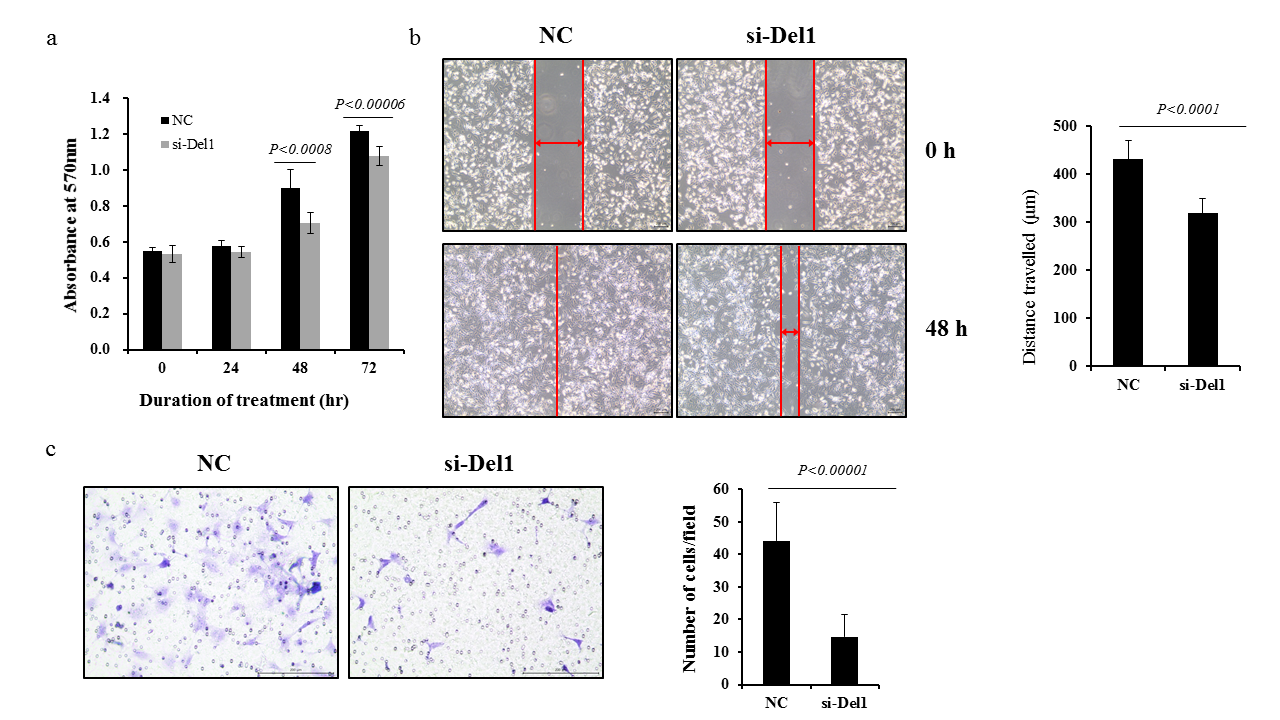

Supplement: Supplementary file 1 [file ijms-20-06162-s001.zip › Supplementary figure 3.tif]

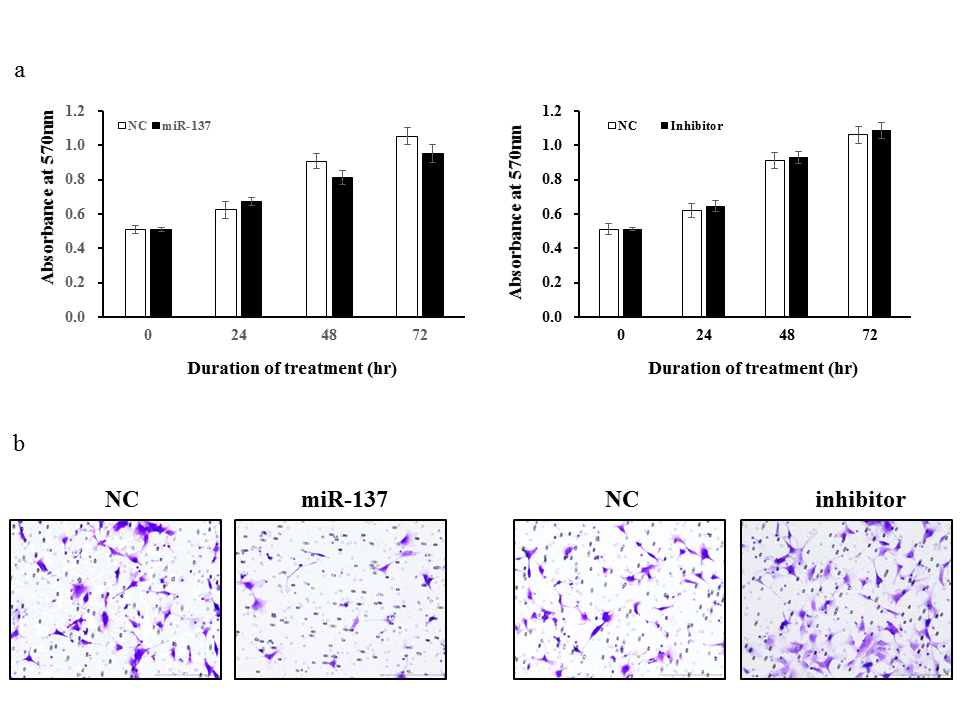

Supplement: Supplementary file 1 [file ijms-20-06162-s001.zip › supplementary figure 1.tif]

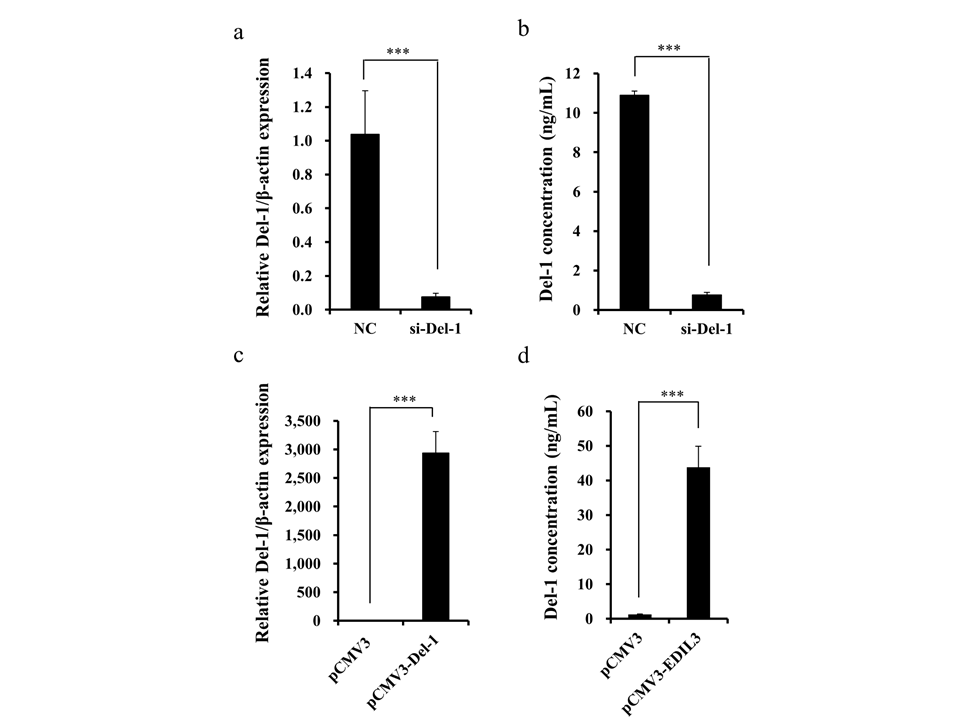

Supplement: Supplementary file 1 [file ijms-20-06162-s001.zip › supplementary figure 2.tif]
